# The latitudinal diversity gradient in South American mammals revisited using a regional analysis approach: The importance of climate at extra-tropical latitudes and history towards the tropics

**DOI:** 10.1371/journal.pone.0184057

**Published:** 2017-09-05

**Authors:** Paula Nilda Fergnani, Adriana Ruggiero

**Affiliations:** Laboratorio Ecotono, Centro Regional Universitario Bariloche-Universidad Nacional del Comahue, INIBIOMA-CONICET, San Carlos de Bariloche, Argentina; Universidad de la Republica Uruguay, URUGUAY

## Abstract

The latitudinal diversity gradient has been considered a consequence of a shift in the impact of abiotic and biotic factors that limit species distributions from the poles to the equator, thus influencing species richness variation. It has also been considered the outcome of evolutionary processes that vary over geographical space. We used six South American mammal groups to test the association of environmental and evolutionary factors and the ecological structuring of mammal assemblages with spatial variation in taxonomic richness (TR), at a spatial resolution of 110 km x 110 km, at tropical and extra-tropical latitudes. Based on attributes that represent what mammal species do in ecosystems, we estimated ecological diversity (ED) as a mean pairwise ecological distance between all co-occurring taxa. The mean pairwise phylogenetic distance between all co-occurring taxa (AvPD) was used as an estimation of phylogenetic diversity. Geographically Weighted Regression analyses performed separately for each mammal group identified tropical and extra-tropical high R^2^ areas where environmental and evolutionary factors strongly accounted for richness variation. Temperature was the most important predictor of TR in high R^2^ areas outside the tropics, as was AvPD within the tropics. The proportion of TR variation accounted for by environment (either independently or combined with AvPD) was higher in tropical areas of high richness and low ecological diversity than in tropical areas of high richness and high ecological diversity. In conclusion, we confirmed a shift in the impact of environmental factors, mainly temperature, that best account for mammal richness variation in extra-tropical regions, whereas phylogenetic diversity best accounts for richness variation within the tropics. Environment in combination with evolutionary history explained the coexistence of a high number of ecologically similar species within the tropics. Consideration of the influence of contemporary environmental variables and evolutionary history is crucial to understanding of the latitudinal diversity gradient.

## Introduction

The Dobzhansky-MacArthur hypothesis (hereafter DMH) [1–3] envisages the latitudinal diversity gradient as the consequence of a shift in the impact of abiotic and biotic factors that limit species distributions from the poles to the equator. Its fundamental proposition suggesting that biotic factors have a more limiting effect in the tropics, whereas the importance of abiotic conditions increases at high latitudes, has been largely explored within the context of range limits (e.g., [4] and references therein). A second proposition of the DMH is that high temperature promotes faster biotic interactions and higher rates of evolution and coevolutionary adaptations in the tropics than in temperate regions [5,6]. This suggests that the latitudinal diversity gradient may be the outcome of evolutionary processes that also vary over geographic space (e.g., [7,8]), and indeed there is some evidence to indicate that diversification rates are faster within the tropics (e.g., [8–10], but see also [11,12]).

Indeed, the DMH is theoretically appealing in accounting for the latitudinal diversity gradient; however, in practice, it has been relatively easier to test it within the context of species range limits and species distributions (e.g., [13]) than for species richness. Although it has been possible to identify the relative contribution of different environmental factors (e.g. temperature, water availability) that limit species richness in tropical and temperate latitudes (e.g., [14–16]), the identification of multiple species interactions that might limit populations, hence scaling up to influence the latitudinal gradient in species richness will be very difficult, if at all possible [5,17]. There is increasing evidence, however, to suggest that ecological differentiation among species contributes to the structuring of the latitudinal gradient in species diversity (e.g., [18–23]). In this study we incorporate information on the ecological diversity (ED; see definition in Methods) of co-occurring species on a local spatial scale of analysis (i.e. within cells of 110 km x 110 km) to address the issue of how differences in the structuring of mammal species assemblages contribute to the high species richness found within the tropics. We also incorporate information on the phylogenetic dimension of diversity (phylogenetic diversity: AvPD) to address the role played by evolutionary history in accounting for differences in mammal species richness between tropical and extra-tropical latitudes.

We extended the first proposition of the DMH, which emphasized the role of temperature as primary determinant of species diversity patterns [5], to other environmental hypotheses often proposed to explain spatial variation in mammal species diversity on a continental scale (ambient energy, water availability, topographic heterogeneity, productivity, climatic variability). We predict that these hypotheses will be better able to account for the variation in mammal species richness within temperate regions than within the tropics. Given that evolutionary history has been shown to have an important effect on species richness patterns, particularly in the tropics [10], we hypothesized that TR is also associated with changes in AvPD, a phylometric closely related to mean phylogenetic distance (MPD: [24]) which has been found to be related to evolutionary time [25]. We have used it here to represent evolutionary history, considering that it may leave a signature on TR patterns [26].

To evaluate how ecological structuring of mammal species assemblages, at a resolution of 110 km x 110 km, influences associations of TR with environmental and evolutionary factors, we identified tropical areas with high TR and low ED as an indication of the coexistence of species with high functional equivalence. We expect that the location of such areas will coincide with regions of high topographic heterogeneity or climatic variability, as environmental heterogeneity should be indispensable in facilitating coexistence through temporal or spatial resource partition among ecologically similar species [27]. On the other hand, we also identified areas of high TR and high ED as an indication of the co-occurrence of specialized species. We predict stronger TR-environment associations within tropical high TR-low ED areas than within tropical high TR-high ED areas. We also analysed whether the proportion of TR variation explained by AvPD in tropical areas harbouring high TR differed between areas with high and low ED, making no a priori predictions.

Throughout the present study we test the environmental predictions of the DMH separately in six South American mammal groups (marsupials, xenarthrans, artiodactyls, carnivores, hystricognaths and primates: [28]), which constitute identifiable cenocrons (sensu [29,30]) with different immigration histories in South America. Autochthonous taxa (xenarthrans and marsupials) dating back to 65 mya [31,32] and mid-Cenozoic immigrants (hystricognath rodents whose earliest records in South America date back to about 41 mya [33] and primates ca. 29 mya [34]) can be considered taxa of tropical affinity, i.e. they faced environmental conditions similar to present-day tropical forests at the time of their arrival in South America. In contrast, the newcomers from North America (artiodactyls and carnivorans) are taxa with savanna-like ecologies that participated in the Great American Biotic Interchange (GABI) that began about 3.8 mya [35] and entered South America during glacial episodes and general climatic cooling [31,33–36]. The history of Cenozoic migration and the evolutionary history of New World mammals is known to have left an imprint on macroecological patterns (body size gradients: [37]), and so might influence species-diversity relationships within tropical and temperate latitudes, as well as the association between richness and functional aspects of diversity. Thus, our regional approach of analysis applied separately to different mammal cenocrons takes this issue into account.

## Methods

### Estimation of the different components of diversity

The spatial patterns of variation in TR, ED and AvPD for each mammal group studied (marsupials, xenarthrans, artiodactyls, carnivorans, hystricognaths and primates) on a continental scale were calculated by Fergnani and Ruggiero [28] at a 110 km x 110 km resolution using an equal area Mollweide projection of the New World in ArcGis 9.2 [38]. Coastal cells that included < 50% of land surface were excluded. The database (available from [28]) included the geographical distribution, functional attributes and phylogeny of 531 mammal species inhabiting North and South America, including marsupials (number of species, N = 78), xenarthrans (N = 29), artiodactyls (N = 29), carnivores (N = 76), hystricognath rodents (N = 191) and primates (N = 128). The taxonomy and geographical distributions of species were taken from NatureServe [39], excluding exotic species and endemic island species.

Taxonomic richness (TR) was the total number of species (marsupials, xenarthrans, artiodactyls, carnivores, hystricognath rodents) or genera (primates) present in each cell of the grid map (details in [28]). Following Harcourt [40], primates were analysed at genera level because the taxonomy at species level is very unstable due to the elevation of subspecies to the level of species (see also, [41]).

Ecological diversity (ED) was an approximation to functional diversity, representing the variety of attributes related to what organisms “do” in their habitat, so as to adopt a more flexible definition of functional diversity that does not require the association of attributes with ecosystem function [42]. Ecological attributes used to calculate ED included different kinds of resources used by species and what species do to acquire them (body mass, home range size, resting or nesting site, substrate use, diet, activity cycle and group size) based on 80 published sources, including books, articles and public databases (see S2 File in [28] for details of the sources and codifications of each attribute). These attributes represent the ecological relationships of mammal species with the environment and have been frequently used in previous analyses of mammal functional diversity (see S2 File in [28] for details). ED was the mean ecological dissimilarity between pairs of coexisting taxa, and was calculated as the mean Gower index [43] in each cell of the grid map, using the FD package in R project [44]. For any pair *(j-k)* of mammal species, the Gower Index (GI_jk_), which ranges from 0 (complete similarity between species pairs) to 1 (complete dissimilarity between species pairs), was calculated as:

$$GI_{jk}=\frac{\sum W_{ijk}S_{ijk}}{\sum W_{ijk}}$$ (1)

Where:

S_ijk_ is the partial similarity coefficient of attribute i for the j-k pair of species.

W_ijk_ is the weight of attribute i for the j-k pair of species. W_ijk_ = 0 if species j and k cannot be compared for attribute i, because either the value of this attribute for species j (X_ij_) or for species k (X_ik_) is unknown (i.e. non-available data for any attribute in any species that is compared). We applied weights to different attributes to ensure that all ecological aspects contributed equally to the ED calculation (see S2 File in [28] for a complete list of weights applied)

For quantitative and ordinal attributes:

$$S_{ijk}=\frac{\left|X_{ij}-X_{ik}\right|}{\text{max}\{X_{i}\}-\text{min}\{X_{i}\}}$$

For the symmetric binary attributes (sensu[45]):

$$\begin{array}{l} S_{ijk}=0\;if\;X_{ij}=X_{ik}=1\\ S_{ijk}=0\;if\;X_{ij}=X_{ik}=0\\ S_{ijk}=1\;if\;X_{ij}\neq X_{ik} \end{array}$$

For the asymmetric binary attributes (sensu[45]):

S_ijk_ formula is the same as for symmetric binary attributes, but:

$$\;W_{ijk}=0\;if\;X_{ij}=X_{ik}=0$$

For nominal attributes:

$$\begin{array}{l} S_{ijk}=0\;if\;X_{ij}=X_{ik}\\ S_{ijk}=1\;if\;X_{ij}\neq X_{ik} \end{array}$$

For each cell we computed GI_jk_ for each combination of species pairs. Then we summed up the values of all combinations of species pairs and divided this number by the total number of species pairs in each cell, to produce the mean GI. The mean Gower index was our estimation of ecological diversity (ED) per cell. It has the property of being independent of the number of species being compared, so it can be used to compare values across grid cells with different species richness.

Phylogenetic diversity (AvPD) was the mean pairwise phylogenetic distance between all taxa in each cell; phylogenetic distance was the sum of the lengths of the branches connecting each pair of species based on the Bininda-Emonds et al. [46] phylogeny. Species not included in this mammal supertree (marsupials: 2, xenarthrans: 0, artiodactyls: 3, carnivorans: 3, hystricognaths: 31, primates: 45) were appended taxonomically as basal polytomies within the clades representing the corresponding genera. Inconsistencies in species names between the phylogeny and NatureServe list were resolved using [47]. For the calculation of AvPD we used the mpd function with cophenetic distance as implemented in the package Picante in R project [48].

Indices calculated by averaging data on pairwise comparisons have been designed to be independent of species richness [49]; we used null models to confirm for each mammal group that the random AvPD and TR were uncorrelated (S1 File). Observed patterns of spatial variation in AvPD and TR are shown in S1 File.

### Choice of environmental variables

The WorldClim v. 1.4 database [50] was used to extract data on environmental variables at a resolution of 30s: TEMP = mean annual temperature, as surrogate for ambient energy [6,15,51]; PREC = annual precipitation, as surrogate for water availability [14,51]; TEMPr = maximum temperature of warmest month—minimum temperature of coldest month and PRECcv = the coefficient of intra-annual variation in precipitation, as surrogates for climatic variability [15,52]; elevation was used to estimate the standard deviation for elevation, a surrogate for topographic heterogeneity (ALTstd) [52–54]. Data on NPP, used to represent primary productivity [55–57], were obtained from the Atlas of the Biosphere (atlas.sage.wisc.edu/) at a resolution of 0.5° x 0.5°, as described in Willmott and Matsuura [58]. We projected all environmental variables onto the Mollweide projection to extract mean values of TEMP, PREC, TEMPr, PRECcv and NPP, or standard deviation values for elevation (ALTstd) for each 110 km x 110 km cell using ArcGIS 9.2 [38].

### Test of the first proposition of DMH

To evaluate whether the role of physical factors that limit species richness increases outside the tropics we applied a regional analysis approach, which implies disaggregation of the global species richness-environment relationships to see how environmental richness predictors perform within tropical and extra-tropical regions. This has raised considerable controversy. On the one hand, it has been suggested that a regional approach may be useful in increasing the descriptive and predictive value of statistical modelling of diversity-environment relationships [59], as well as identifying missing predictor variables [60]; however, it has also been viewed as precluding the test of hypotheses and model predictions as can be done with global statistics [61]. Nonetheless, regional and global approaches to the analysis of diversity-environment relationships can both be informative, provided global and regional predictions derived from environmental hypotheses are warranted [62,63], and thus we think a regional analysis is most appropriate to evaluate predictions of the DMH.

Our regional analysis approach followed several steps:

1. Quantification of regional species diversity–environment and evolutionary associations: We assessed TR-environment associations using Geographically Weighted Regression analysis (GWR) as applied in SAM [64]. For each cell on the grid map, GWR obtained an intercept, *b* coefficients for each predictor variable, and a local adjusted R^2^ using an adaptive Gaussian kernel with a bandwidth that included the nearest 10% neighbours. The adaptive kernel ensured that the parameters obtained for focal cells located close to borders of the continent were based on the same number of observations as for local cells located away from borders. The use of 10% neighbours was a conservative decision that allowed use of a relatively high number of cells for accurate estimation of the parameters, while still allowing the detection of regional variation patterns in species-environment relationships.
  While examining the associations of TR with environment, we hypothesized that AvPD may leave a signature on TR patterns [26], and so it was included as a additional explanatory variable in the statistical models tested to account for the spatial variation in TR.
2. Identification of high R^2^ areas within tropical and extra-tropical regions: We identified the quartiles of distribution of the local R^2^ obtained from GWR as a function of all environmental and evolutionary predictors. We mapped the quartiles of distribution of the local R^2^ to identify areas with the highest local R^2^ (i.e. R^2^ values included in the upper quartile) that indicated regions where the geographical variation in TR was strongly explained by the predictors. A posteriori, we classified high R^2^ areas as “tropical” if they were located between 23° N and 23°S, and “extra-tropical” if they were located at higher northern and southern latitudes.
  Our analytical approach differs from previous studies that tested predictions for the latitudinal diversity gradient by distinguishing “a priori” tropical vs extra-tropical regions. These regions are not climatically homogeneous. Although the tropics is climatically more stable than the extra-tropics it cannot be considered a “single phenomenon” [65]. Thus, we let the data themselves distinguish those areas where TR-environment and evolutionary relationships were strong enough to warrant regional comparisons between tropical and extra-tropical areas where the proportion of TR variation explained by environment and AvPD was indeed biologically meaningful. Nonetheless, our classification based on the R^2^ quartiles is representative of previously described environment-richness relationships for tropical and temperate regions [14].
3. Analysis of b values: To verify the prediction of a higher effect of environment on diversity in the extra-tropics than in the tropics, we first averaged the local *b* values obtained by GWR, for each predictor in each cell, over the total number of cells encompassed within each high R^2^ area. We took into account differences in the size of high R^2^ areas to reach conclusions as to the role of environment in tropical and extra-tropical latitudes. We used mean *b* values for each variable and the number of cells in each high R^2^ area to estimate, for tropical and extra-tropical latitudes, a common measure of effect size (Fisher’s Z-transfrom, Zr: Hedges and Olkin [66]) using MetaWin v.2 [67].

### Test of the association between the ecological structuring of mammal species assemblages and species richness

To test whether environmental effects on TR were stronger in tropical areas of high TR-low ED than tropical areas of high TR-high ED we followed several steps:

1. Identification of tropical areas with high TR and high or low ED: We mapped the upper quartiles of the TR distribution and the upper and lower quartiles of the ED distribution. Subsequent analyses were restricted to tropical areas with high TR-high ED (i.e., containing the values in the upper quartiles of the TR and ED distributions) and high TR-low ED (i.e., containing values in the upper quartile of the TR distribution and lower quartile of the ED distribution).
2. Partial regression analysis: We conducted a partial regression analysis [68] to separate TR variation in each cell into (a) environmental effects: fraction of TR variation explained by environmental descriptors, independently of AvPD; (b) phylogenetically structured environmental variation: proportion of TR variation explained by the joint effect of phylogeny and environment; (c) phylogenetic effects: fraction of TR variation explained by AvPD independently of the environmental variables; and (d) unexplained variation: proportion of TR variation explained neither by the AvPD nor by any of the environmental variables included in our analysis. Each component of variation was estimated for each cell, and then averages were obtained over all high TR-high ED and high TR-low ED areas. The DMH predicts that environmental effects (represented by fractions (a) and (b)) will be stronger in high TR-low ED than in high TR-high ED areas.
3. To further analyse differences in the explanatory capacity of the environment to account for TR variation across tropical areas, we evaluated whether climatic variability and topographic heterogeneity were higher in high TR-low ED areas than in high TR-high ED areas.

## Results

### TR-environment and evolutionary associations in tropical and extra-tropical areas

**Fig 1:**
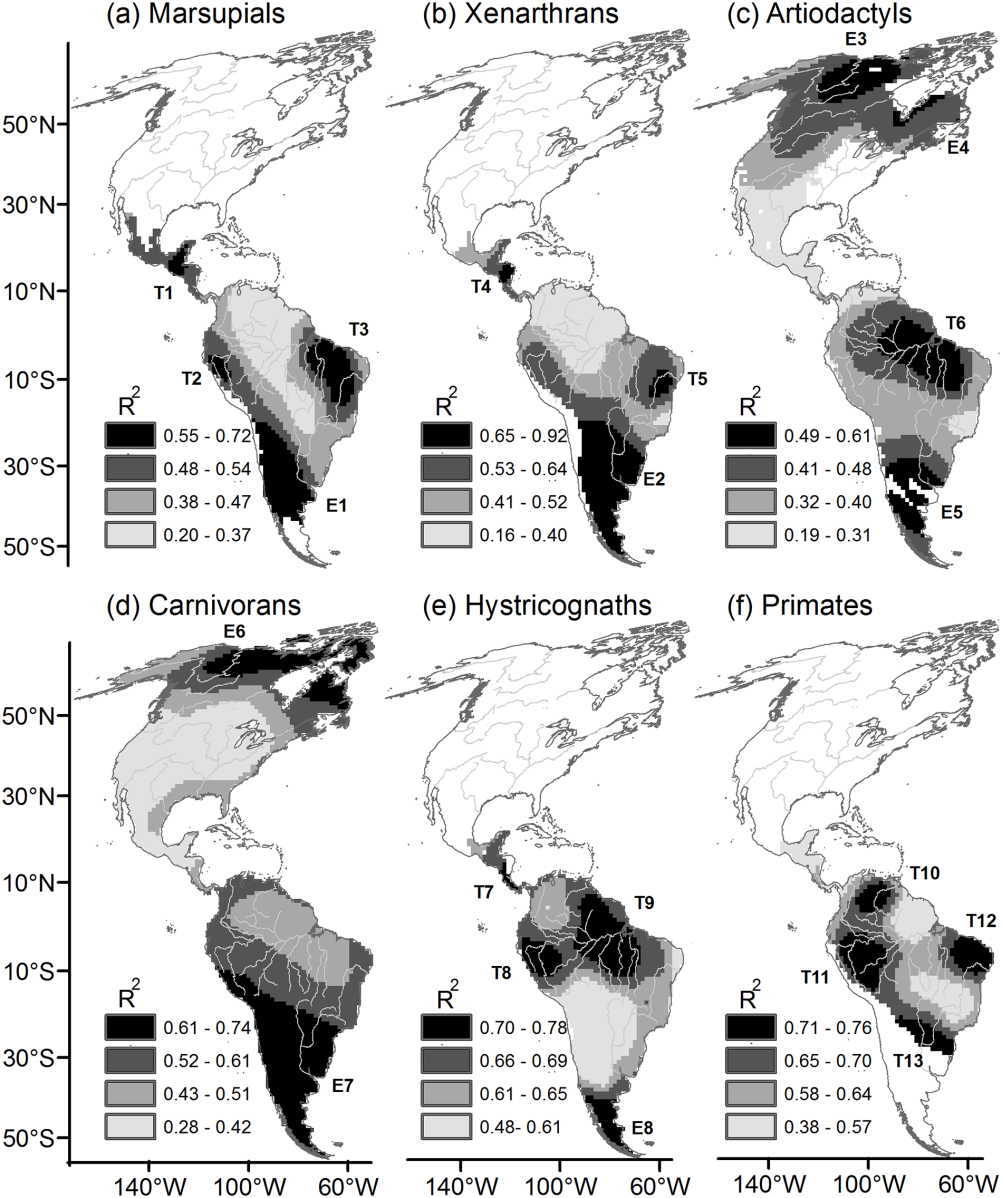
Local coefficients of determination (R^2^) obtained from geographically weighted regression (GWR), indicating the proportion of variation in taxonomic richness (TR) accounted for by the environmental predictors and phylogenetic diversity (AvPD). Local R^2^ values are displayed according to quartiles; high R^2^ areas corresponded to the upper quartiles shown as black areas. T = tropical, E = extra-tropical. Maps are in Mollweide equal-area projection.

Physical and climatic factors that limit TR have high explanatory power, thus leading to high R^2^ at temperate latitudes in southern South America and at high latitudes in northern North America. However, high R^2^ areas were also identified at tropical latitudes within the Amazon Basin (artiodactyls, hystricognaths, primates Fig 1c, 1e and 1f), the Caatinga and Cerrado of northeastern Brazil (marsupials, xenarthrans, primates, Fig 1a, 1b and 1f) and Central America (marsupials, xenarthrans, hystricognaths, Fig 1a, 1b and 1e).

**Fig 2:**
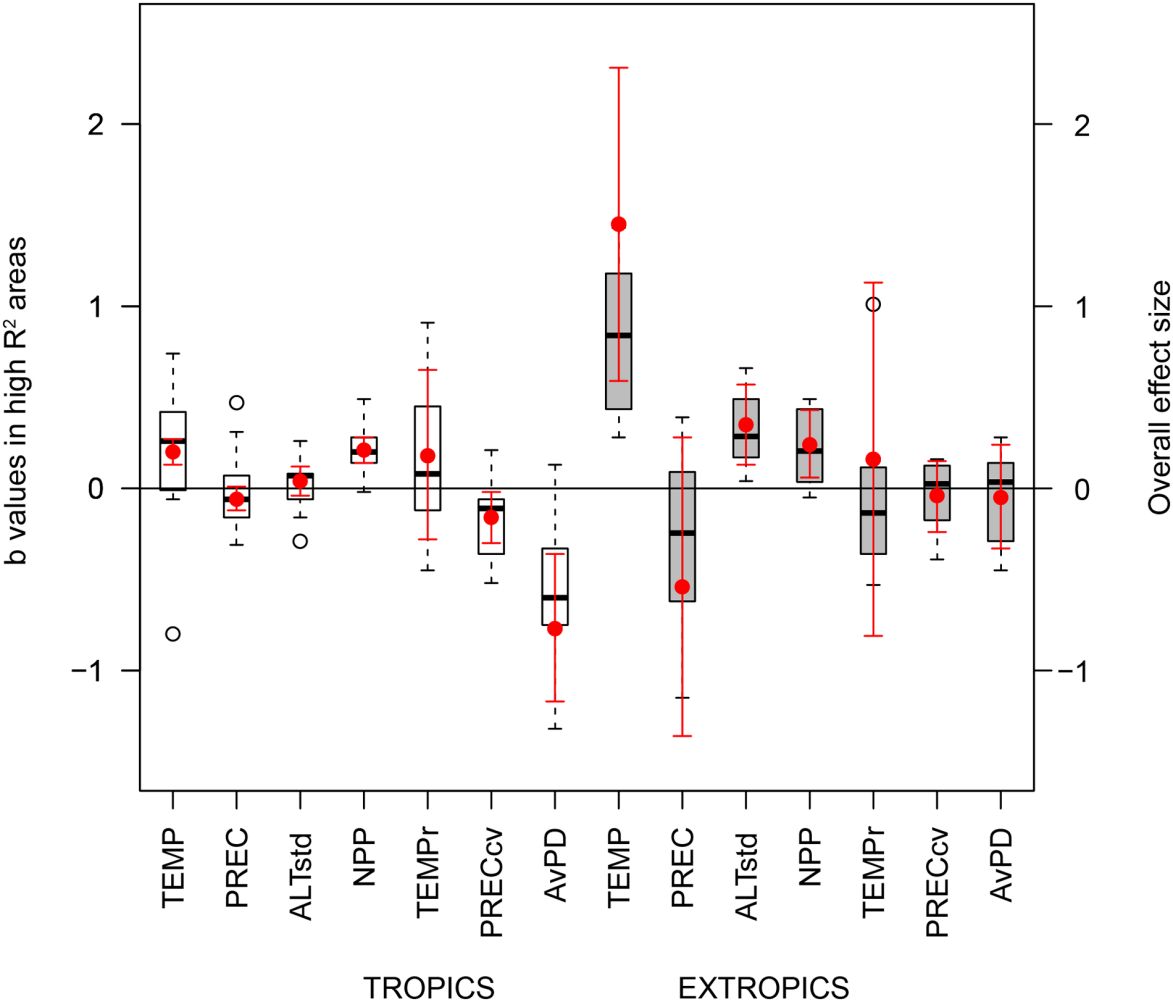
Plot of *b* values and overall effect sizes of environmental variables and phylogenetic diversity (AvPD) on taxonomic richness (TR), quantified in tropical and extra-tropical high R^2^ areas. The box plot shows the distribution of mean *b* values in high R^2^ areas. For each high R^2^ area depicted in Fig 1 we calculated the mean *b* value as the average *b* values obtained in GWR for each high R^2^ 110 km x 100 km cell. The complete list of mean *b* values is in S2 File. The red dots are the overall effect sizes and the red bars indicate their confidence interval. Overall effect sizes (Fisher´s Z-transfrom, Zr: [66]) took into account differences in the size of high R^2^ areas, and were estimated using MetaWin v.2 [67]. Beta coefficients greater than 1 were set at 0.99 for the calculations of effect sizes. TEMP = mean annual temperature, PREC = annual precipitation, ALTstd = standard deviation in elevation, TEMPr = temperature range, PRECcv = the coefficient of intra-annual variation in precipitation, AvPD = phylogenetic diversity.

We found a greater overall effect of TEMP (Zr = 1.45), PREC (Zr = -0.54) and ALTstd (Zr = 0.35) on TR across extra-tropical high R^2^ areas than across tropical high R^2^ areas where overall environmental effects ranged from Zr = 0.06 to 0.21 (Fig 2, S2 File). Across tropical high R^2^ areas, TEMP effects were highly variable, including highly positive (b > 0.5 in xenarthrans and artiodactyls), highly negative (b = -0.8, marsupials) or weak associations with TR (Fig 2, S2 File). AvPD was the most important variable to account for TR variation across tropical high R^2^ areas (Zr = -0.77; Fig 2, S2 File). In contrast, the overall effect of AvPD was very low (Zr = -0.05) across extra-tropical high R^2^ areas (Fig 2, S2 File).

### TR-environment associations in tropical areas of high TR and high or low ED

**Fig 3:**
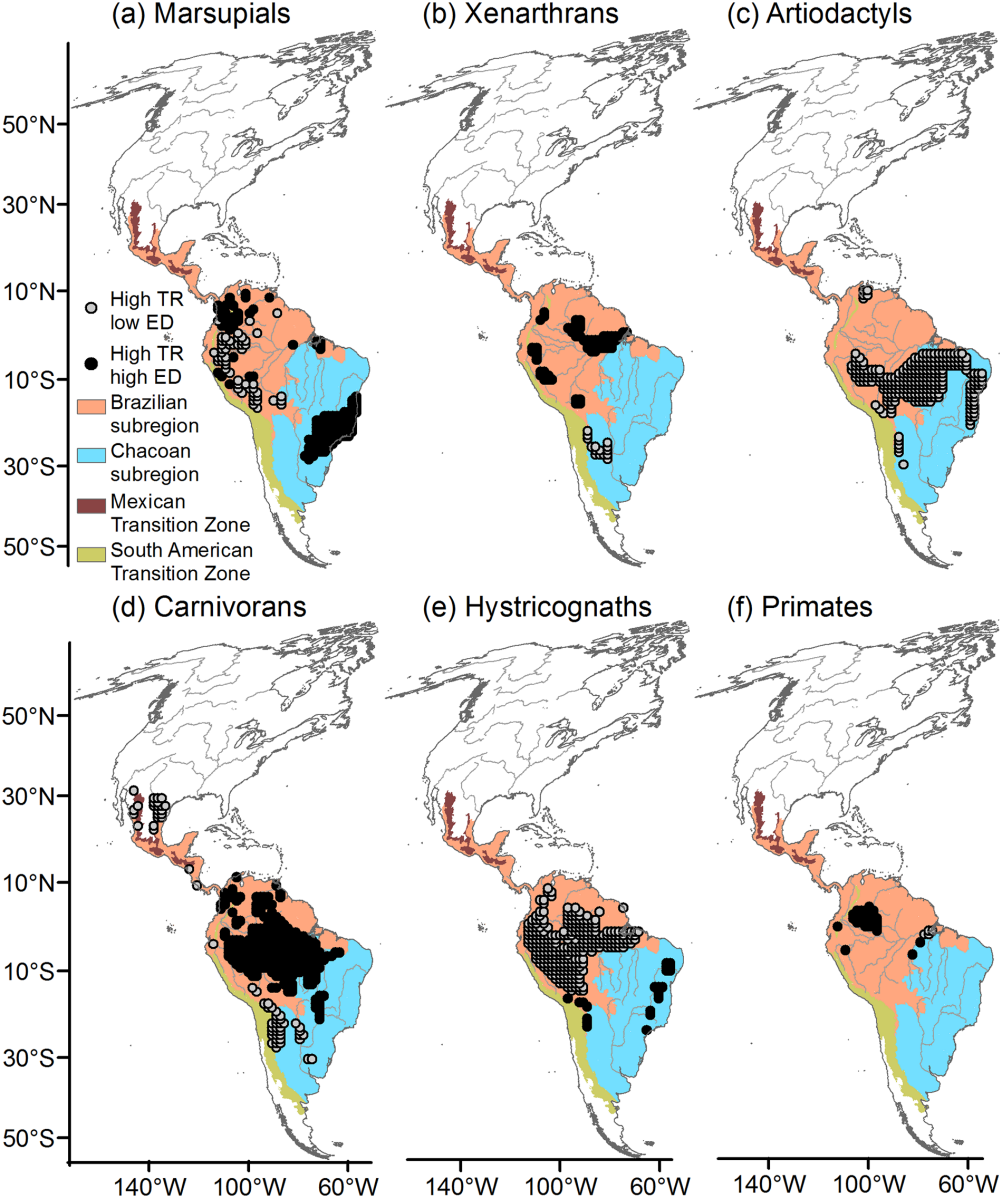
Areas of high taxonomic richness and high ecological diversity (high TR-high ED), and high taxonomic richness and low ecological diversity (high TR-low ED) identified within tropical latitudes. Black circles: High TR-high ED; grey circles: High TR-low ED. The biogeographic regionalization of the Neotropical region is shown [69,70]. Maps are in Mollweide equal-area projection.

We identified high TR-high ED and high TR-low ED areas within tropical latitudes for marsupials, xenarthrans, carnivorans hystricognaths and primates (Fig 3a, 3b and 3d–3f). Artiodactyls only showed high TR-low ED areas within the tropics (Fig 3c); the areas in primates corresponded to a low number of cells (Fig 3f). Thus, artiodactyls and primates were excluded from further analyses.

Contrary to our original prediction, the location of tropical high-TR low ED areas was not associated consistently across taxa with areas of high topographical (ALTstd) and climatic (TEMPr and PRECcv) heterogeneity (Table 1, S3 File). Nonetheless, environmental factors, independently of, or in combination with AvPD, did explain a greater proportion of TR variation in high TR-low ED than in high TR-high ED areas in all mammal groups, particularly for xenarthrans (see the totals of a + b in Table 2).

**Table 1:** Mean values ± standard deviation of topographic and climatic heterogeneity in tropical areas of high taxonomic richness and high ecological diversity (high TR-high ED) and high taxonomic richness and low ecological diversity (high TR-low ED).

	marsupials		xenarthrans		carnivorans		hystricognaths	
	High TR-high ED	High TR-low ED	High TR-high ED	High TR-low ED	High TR-high ED	High TR-low ED	High TR-high ED	High TR-low ED
ALTstd	205±237	174±312	127±202	208±384	84±163	409±313	330±346	94±193
TEMPr	167±36	133±28	119±19	265±15	136±29	252±49	184±35	123±18
PRECcv	53±22	36±19	42±13	63±18	52±18	75±23	78±16	43±16
N	132	54	65	15	132	63	22	275

Topographic heterogeneity is represented by ALTstd (standard deviation of elevation). Climatic variability is represented by TEMPr (temperature range) and PRECcv (coefficient of variation in precipitation). N: number of cells. We predicted that high TR-low ED areas would coincide with regions of higher topographic and/or climatic heterogeneity than high TR-high ED areas.

**Table 2:** Partial regression analysis of variation in taxonomic richness (TR) in tropical areas of high taxonomic richness and high ecological diversity (high TR-high ED) and high taxonomic richness and low ecological diversity (high TR-low ED).

Components of variation	marsupials		xenarthrans		carnivorans		hystricognaths	
	High TR-high ED	High TR-low ED	High TR-high ED	High TR-low ED	High TR-high ED	High TR-low ED	High TR-high ED	High TR-low ED
Environment (a)	30	35	24	36	24	35	23	8
Environnment—AvPD (b)	12	10	0	34	24	17	18	39
AvPD (c)	0	0.1	15	3	3	4	19	21
Unknown (d)	57	55	61	26	49	44	40	31

^(a)^ Fraction of TR variation explained by the environmental descriptors independently of phylogenetic diversity (AvPD).

^(b)^ Fraction of TR variation explained by the joint effect of phylogeny and environment.

^(c)^ Fraction of TR variation explained by AvPD independently of environmental variables.

^(d)^ Unexplained variation. Each component of variation was estimated for each cell, and the table shows the mean values for each component of TR variation (a, b, c, d), averaged over all cells in high TR-high ED and high TR-low ED areas, expressed as percentages. We predicted that environment-TR associations would be stronger in high TR-low ED areas than in high TR-high ED areas, then fractions a + b will be higher in high TR-low ED than in high TR-high ED areas

We found differences across mammal groups in the extent to which AvPD, either independently of or in combination with environment, influences TR patterns within high TR-low ED areas. In xenarthrans and hystricognaths, a high proportion of TR variation in high TR-low ED areas was explained by AvPD in combination with environment (xenarthrans: 34%; hystricognaths: 39%; Table 2); the proportion of TR variation explained by AvPD independently of environment was higher for hystricognaths (21%) than for xenarthrans (3%). In contrast, the TR variation of marsupials and carnivorans in high TR-low ED areas was mainly accounted for by environmental factors, independently of or in combination with AvPD (Table 2).

In high TR-high ED areas, AvPD explained a low proportion (< 30% including fractions b + c in Table 2) of TR variation in the majority of mammal groups, except for hystricognaths and carnivores (Table 2).

## Discussion

### Climate is more important in accounting for mammal species richness patterns in extra-tropical latitudes than within the tropics

Our study verified stronger environmental effects on TR on a regional scale within extra-tropical latitudes than within the tropics, thus verifying the first proposition of the DMH [5]. We confirmed that energy is a more important driver of diversity at higher latitudes than within the tropics [5,14,71,72]. This suggests the predominant role of the ambient energy hypothesis (represented by TEMP) over the productivity, heterogeneity and climatic variability hypotheses in accounting for regional differences in TR between tropical and extra-tropical latitudes in America. Our results agree with previous evidence showing that temperature strongly influences mammal richness on a global scale [11], and it is the principal environmental correlate of caviomorph species richness in South America [73]. However, we contradict previous findings suggesting that water-related variables (PREC and NPP) and topographic heterogeneity best predicted richness in low-latitude, high-energy regions [14,16]. Indeed, the present study found a low overall positive effect of NPP on richness in both tropical and extra-tropical latitudes, in apparent contradiction with the idea that primary productivity is the primary correlate of mammal species richness patterns in South America [53,74], and on a global scale [25]. The low association between NPP and TR found in the present study is hardly an artefact produced by the effect of introducing multicollinearity into the analyses [75]; geographically weighted regression (GWR) has in fact proved to be very robust to the effects of multicollinearity [76] and local VIFs obtained for variables in high R^2^ areas in all GWR models fitted to our data were below the threshold of 10 [77]; S4 File). Rather, the low TR-NPP association emerged after taking into account the effects of phylogenetic diversity and considering non-stationarity in TR-environment relationships, which could influence the perception of species richness-environment relationships. A worldwide analysis showed that the effect of productivity (AET) on mammal species richness shows varying importance across regions [60]. Belmaker & Jetz [11] examined the effects of NPP on mammal species richness across different bioregions of the world, and also found lack of support for a direct association of NPP with richness, or an indirect association mediated through niche diversity. Our study also suggests that the effect of productivity on South American mammal species richness may be highly variable on a regional scale.

### Evolutionary history is important in accounting for species richness patterns within the tropics

We found that phylogenetic diversity (AvPD) explained a high proportion of the variation in species richness in tropical latitudes, suggesting that evolutionary history is crucial to the explanation of species richness patterns towards the tropics. Null models demonstrated that the associations between AvPD and TR are biologically meaningful because these two variables are not inherently correlated by design. The negative AvPD-TR relationship found within the tropics suggests that rapid and/or recent in situ diversification after a few long-distant migration events may underlie the structuring of local species assemblages showing high richness and low phylogenetic diversity [26,78,79]. It has been proposed that during the Great American Biotic Interchange (GABI) that began about 3.8 mya [35] there was a decline in the number of endemic South American mammal families, although overall generic mammal diversity remained stable and the total number of genera increased by diversification of taxa from North American immigrants [80]. The negative AvPD-TR relationship could represent the predominance of young mammal families within the tropics, leading to mammal species assemblages composed of a few basal families and a large number of more recent families [81]. In our data set there are only four mammal families (Myrmecophagidae, Ursidae, Didelphidae and Dasypodidae) that originated prior to ca. 48 Ma, before the Eocene–Oligocene climate shift, which contributed to the richness of tropical latitudes [46]; the remaining mammal families recorded in the tropics were in clades aged between 4.8 and 22 Ma, according to [46].

It has been suggested that high mammal species richness within the tropics is the consequence of high diversification rates [8,10], but see also [12,22]. High levels of speciation in the tropics along with the existence of phylogenetic niche conservatism has been proposed as an explanation for the pattern of tropical mammal communities showing high species richness and functional redundancy [82]. The phylogenetic conservatism of dominant ecological characters found across many lineages [83] explains the tendency for closely related species to be more ecologically similar than would be expected if no deterministic processes were affecting ecological evolution [84], thus leading closely related species to inhabit rather more similar environments than distantly related ones (phylogenetic niche conservatism hypothesis: [83–85]). Safi et al. [82] suggested that the pattern of tropical mammal communities showing high species richness and functional redundancy may have resulted from high levels of speciation in the tropics, producing many species of conserved niches that maintained a high level of functional similarities while staying within or near their ancestral geographical range. The hypothesis of niche conservatism was also implied in analysis of bat diversity patterns across South America [86]. The occurrence of high TR-low ED areas within tropical latitudes found in our study is consistent with this interpretation; however, our study suggests that for each mammal group studied separately the occurrence of high mammal richness and functional redundancy is rather a geographically restricted phenomenon in the tropics, except for the hystricognaths and artiodactyls (see also [28]), contrasting with the ubiquitous pattern shown for the whole mammal assemblage on a global scale [11,82]. Future studies are needed to evaluate the extent to which the pattern shown for the whole mammal assemblage in South America [11,82] is due to the influence of rodents due to their high number of species.

Contrary to our original prediction, at a spatial resolution of 110 km x 110 km in high richness tropical regions, high topographical and climatic heterogeneity did not account for the packing of ecologically similar species in all mammal groups except for xenarthrans and carnivores. The presence of the Andes is a major geofeature, promoting an increase in environmental heterogeneity towards the west of the continent, along with a northwest-south east arid diagonal in South America formed after the establishment of the present climatic zonation [87], which at present represents the transition between the Neotropical and Andean biogeographic regions [70]. In xenarthrans and carnivores alone, the location of areas of high richness and functional redundancy overlapped regions of high topographic relief and climatic variability, or were close to biogeographical transitions (Figs S4-S6 in S3 File). At least in canids, a process of rapid colonization and speciation in South America was associated with marked changes in morphology, although these were unrelated to phylogeny, which allowed a rapid spread of the species on the continent, occupying different climates, which suggests that climatic heterogeneity was a key factor in their ecological diversification [88]. This also supports the idea that recent colonization events in combination with fast adaptive radiation and high interspecific competition account for high levels of functional diversity and low levels of phylogenetic diversity in mammals (competition hypothesis: Safi et al. 2011). Thus, the present study offers evidence to suggest that at a 110 km x 100 km grain, climatic and topographic variability is only partially involved in the ecological structuring of tropical mammal communities of high richness and functional redundancy. Our findings are consistent with previous evidence suggesting that topographic heterogeneity, either independently of, or in combination with vegetation and climate, plays only a minor role in explaining mammal species richness in the Neotropics [89], and the more general notion that habitat heterogeneity plays a secondary role in accounting for richness patterns on a large geographical scale [90].

Hystricognaths, marsupials and artiodactyls showed co-occurrence of ecologically similar species in high richness areas within the Amazonian Basin where topographical and climatic heterogeneity is low, at least on a macro-scale of analysis, thus complementing previous analyses showing high diversity of mammals and other vertebrate groups in the Amazon [73,91]. This pattern could reflect the existence of tropical regions where mammal communities are not saturated, and where competition, although important in limiting the evolution of resource-related traits, may not limit the co-occurrence of species with similar traits [19]. There are examples where the appearance of “open niches” represents ecological opportunities for trait divergence and radiation, leading to an accumulation of sympatric species sharing similar resource-related traits (e.g., hylid treefrogs [19]).

In support of our original prediction, contemporary environment, either independently or combined with evolutionary history, accounted for a greater proportion of the variation in the richness of marsupials, xenarthrans, carnivorans and hystricognaths within tropical areas of high richness and low ecological diversity (high TR-low ED areas, Table 2), than within tropical areas of high richness and high ecological diversity (high TR-high ED areas, Table 2); however, a considerable proportion of variation in TR remained unexplained by our statistical models, as previously found for the whole South American mammal fauna on a continental scale [53,89]. In xenarthrans and hystricognaths, environmental factors in combination with evolutionary history were more important to account for TR variation within areas of high richness and low ecological diversity, than in areas of high richness and high ecological diversity. This suggests that the occurrence of tropical assemblages of high richness and functional redundancy in these two mammal groups, representing ancient and mid-Cenozoic cenocrones, is phylogenetically structured, and may be associated with major paleoenvironmental changes that occurred during their evolutionary history in South America [80,92], which is known to have influenced the evolution of key functional traits [92,93]. For example, it has been documented that during the latest part of the Cenozoic many xenarthrans followed a similar dental modification, from low-crowned to high-crowned morphology, in association with the general climatic trends that promoted a change from predominantly closed-forested, warm and wet habitats to open temperate grasslands, to hot deserts and cold habitats [92,93].

In the hystricognaths, the appearance of vast zones of inundated forests (“varzea”) in the Amazonian Basin, which occurred at about 14 Ma, promoted speciation events in association with the appearance of “arboreality” as a key innovation in the evolutionary history of echimyids [94]. Mainly due to their poor dispersal capacities and small geographic ranges, rodent lineages tend to have fragmented populations that could experience reduced gene flow, thus increasing opportunities for in situ speciation [95]. The complexity of tropical forests [80] could actually be sufficient to generate the fine-scaled habitat heterogeneity necessary to accommodate high diversity in small mammals of high functional equivalence, as they presumably would have had more ways to partition microhabitat than large mammals. Thus, for instance, the co-occurrence of similar arboreal species on a large-geographical scale could be accounted for by the partition of occupancy on finer spatial scales, for instance, by filling different kinds of forests, e.g. genera mainly related to varzea, bamboo patches or mature primary forests [94].

In conclusion, our study shows the added value of considering a regional analysis approach and multi-dimensional aspects of species diversity to test the relative contribution of environmental and evolutionary factors to species richness patterns. We found that environmental variation does indeed account for a greater proportion of mammal species richness outside the tropics; however, environment and evolutionary history (here represented by phylogenetic diversity) also accounts for considerable variation in taxonomic richness within the tropics. Environmental and historical factors also participate in the functional structuring of mammal species assemblages within the tropics, which reaffirms the idea that the integration of contemporary environmental variables and evolutionary history is crucial for a comprehensive understanding of the latitudinal diversity gradient.

## Supporting information

S1 File Null models to evaluate the association between phylogenetic diversity (AvPD) and taxonomic richness (TR), and observed spatial patterns of variation in AvPD and TR. (DOC)

S2 File Slope (*b)* values and overall effect sizes of environmental variables and phylogenetic diversity (AvPD) on richness obtained for high R^2^ areas. (DOC)

S3 File The mapping of tropical areas with high taxonomic richness and low ecological diversity (high TR-low ED) and high taxonomic richness and high ecological diversity (high TR-high ED), and variation in topographic and climatic heterogeneity. (DOC)

S4 File Evaluation of collinearity in geographically weighted regression (GWR) models. (DOC)

## References

[1] Brown JH (1995) Macroecology. Chicago: University of Chicago Press.

[2] Dobzhansky T (1950) Evolution in the tropics. American Scientist 38: 209–221.

[3] MacArthur RH (1972) Geographical ecology: patterns in the distribution of species: Princeton University Press.

[4] Cunningham HR, Rissler LJ, Buckley LB, Urban MC (2016) Abiotic and biotic constraints across reptile and amphibian ranges. Ecography 38: 1–8.

[5] Brown JH (2014) Why are there so many species in the tropics? Journal of Biogeography 41: 8–22. doi: 10.1111/jbi.12228 25684838 10.1111/jbi.12228 PMC4320694

[6] Rohde K (1992) Latitudinal gradients in species diversity: the search for the primary cause. Oikos 65: 514–527.

[7] Marin J, Hedges SB (2016) Time best explains global variation in species richness of amphibians, birds and mammals. Journal of Biogeography.

[8] Machac A, Graham CH, Steppan SJ, Bronstein JL (2017) Regional Diversity and Diversification in Mammals. The American Naturalist 189: E000–E000. 10.1086/689398 28035892

[9] Mittelbach GG, Schemske DW, Cornell HV, Allen AP, Brown JM, Bush MB, et al (2007) Evolution and the latitudinal diversity gradient: speciation, extinction and biogeography. Ecology Letters 10: 315–331. doi: 10.1111/j.1461-0248.2007.01020.x 17355570 10.1111/j.1461-0248.2007.01020.x

[10] Rolland J, Condamine FL, Jiguet F, Morlon H (2014) Faster speciation and reduced extinction in the tropics contribute to the mammalian latitudinal diversity gradient. PLoS Biol 12: e1001775 doi: 10.1371/journal.pbio.1001775 24492316 10.1371/journal.pbio.1001775 PMC3904837

[11] Belmaker J, Jetz W (2015) Relative roles of ecological and energetic constraints, diversification rates and region history on global species richness gradients. Ecology Letters 18: 563–571. doi: 10.1111/ele.12438 25919478 10.1111/ele.12438

[12] Weir JT, Schluter D (2007) The latitudinal gradient in recent speciation and extinction rates of birds and mammals. Science 315: 1574–1576. doi: 10.1126/science.1135590 17363673 10.1126/science.1135590

[13] Kaufman DM (1995) Diversity of New World mammals: universality of the latitudinal gradients of species and bauplans. Journal of Mammalogy 76: 322–334.

[14] Hawkins BA, Field R, Cornell HV, Currie DJ, Guégan JF, Kaufman DM, et al (2003) Energy, water, and broad-scale geographic patterns of species richness. Ecology 84: 3105–3117.

[15] Currie DJ (1991) Energy and large-scale patterns of animal-and plant-species richness. The American Naturalist: 27–49.

[16] Kerr JT, Packer L (1997) Habitat heterogeneity as a determinant of mammal species richness in high-energy regions. Nature 385: 252–254.

[17] Schemske DW, Mittelbach GG, Cornell HV, Sobel JM, Roy K (2009) Is there a latitudinal gradient in the importance of biotic interactions? Annu Rev Ecol Evol Syst 40: 245–269.

[18] Lamanna C, Blonder B, Violle C, Kraft NJ, Sandel B, Šímová I, et al (2014) Functional trait space and the latitudinal diversity gradient. Proceedings of the National Academy of Sciences 111: 13745–13750. 10.1073/pnas.1317722111 PMC4183280 25225365

[19] Wiens JJ (2011) The niche, biogeography and species interactions. Philosophical Transactions of the Royal Society B: Biological Sciences 366: 2336–2350. 10.1098/rstb.2011.0059 PMC3130432 21768150

[20] Belmaker J, Sekercioglu CH, Jetz W (2012) Global patterns of specialization and coexistence in bird assemblages. Journal of Biogeography 39: 193–203.

[21] Cardillo M (2002) The life-history basis of latitudinal diversity gradients: how do species traits vary from the poles to the equator? Journal of Animal Ecology 71: 79–87.

[22] Schluter D (2016) Speciation, Ecological Opportunity, and Latitude: (American Society of Naturalists Address). The American Naturalist 187: 1–18. doi: 10.1086/684193 26814593 10.1086/684193

[23] Davies TJ, Meiri S, Barraclough TG, Gittleman JL (2007) Species co-existence and character divergence across carnivores. Ecology letters 10: 146–152. doi: 10.1111/j.1461-0248.2006.01005.x 17257102 10.1111/j.1461-0248.2006.01005.x

[24] Webb CO (2000) Exploring the phylogenetic structure of ecological communities: an example for rain forest trees. The American Naturalist 156: 145–155. doi: 10.1086/303378 10856198 10.1086/303378

[25] Oliveira BF, Machac A, Costa GC, Brooks TM, Davidson AD, Rondinini C, et al (2016) Species and functional diversity accumulate differently in mammals. Global Ecology and Biogeography: doi: 10.1111/geb.12471

[26] Fritz SA, Rahbek C (2012) Global patterns of amphibian phylogenetic diversity. Journal of biogeography 39: 1373–1382.

[27] MacArthur RH (1958) Population ecology of some warblers of northeastern coniferous forests. Ecology 39: 599–619.

[28] Fergnani PN, Ruggiero A (2015) Ecological diversity in South American mammals: their geographical distribution shows variable associations with phylogenetic diversity and does not follow the latitudinal richness gradient. PLoS ONE 10: e0128264 doi: 10.1371/journal.pone.0128264 26053742 10.1371/journal.pone.0128264 PMC4460121

[29] Morrone JJ (2010) Evolutionary Biogeography: Principles and Methods In: Gailis M, Kalnins S, editors. Biogeography: Nova Science Publishers, Inc pp. 1–62.

[30] Reig OA (1962) Las interacciones cenogenéticas en el desarrollo de la fauna de vertebrados tetrápodos de América del Sur. Ameghiniana 1: 131–140.

[31] Delsuc F, Vizcaíno SF, Douzery EJP (2004) Influence of Tertiary paleoenvironmental changes on the diversification of South American mammals: a relaxed molecular clock study within xenarthrans. BMC Evolutionary Biology 4: 13. 15115541 10.1186/1471-2148-4-11 PMC419338

[32] Springer MS, Meredith RW, Janecka JE, Murphy WJ (2011) The historical biogeography of Mammalia. Philosophical Transactions of the Royal Society of London B: Biological Sciences 366: 2478–2502. doi: 10.1098/rstb.2011.0023 21807730 10.1098/rstb.2011.0023 PMC3138613

[33] Antoine P-O, Marivaux L, Croft DA, Billet G, Ganerød M, Jaramillo C, et al (2012) Middle Eocene rodents from Peruvian Amazonia reveal the pattern and timing of caviomorph origins and biogeography. Proceedings of the Royal Society of London Series B: Biological Sciences 279: 1319–1326. doi: 10.1098/rspb.2011.1732 21993503 10.1098/rspb.2011.1732 PMC3282368

[34] Perez SI, Tejedor MF, Novo NM, Aristide L (2013) Divergence times and the evolutionary radiation of New World monkeys (Platyrrhini, Primates): an analysis of fossil and molecular data. PLoS ONE 8: e68029 doi: 10.1371/journal.pone.0068029 23826358 10.1371/journal.pone.0068029 PMC3694915

[35] Pascual R (2006) Evolution and Geography: The Biogeographic History of South American Land Mammals. Annals of the Missouri Botanical Garden 93: 209–230.

[36] Woodburne MO (2010) The Great American Biotic Interchange: dispersals, tectonics, climate, sea level and holding pens. Journal of Mammalian Evolution 17: 245–264. doi: 10.1007/s10914-010-9144-8 21125025 10.1007/s10914-010-9144-8 PMC2987556

[37] Morales-Castilla I, Olalla-Tarraga MA, Purvis A, Hawkins BA, Rodriguez MA (2012) The imprint of Cenozoic migrations and evolutionary history on the biogeographic gradient of body size in New World mammals. The American Naturalist 180: 246–256. doi: 10.1086/666608 22766934 10.1086/666608

[38] ESRI (2007) ArcGIS 9.2 software. Redlands, CA.

[39] Patterson BD, Willig MR, Stevens RD (2003) Trophic strategies, niche partitioning, and patterns of ecological organization In: Kunz TH, Fenton MB, editors. Bat Ecology Chicago: University of Chicago Press pp. 536–579.

[40] Harcourt AH (2006) Rarity in the tropics: biogeography and macroecology of the primates. Journal of biogeography 33: 2077–2087.

[41] Groves C (2004) The what, why and how of primate taxonomy. International Journal of Primatology 25: 1105–1126.

[42] Petchey OL, Gaston KJ (2006) Functional diversity: back to basics and looking forward. Ecology Letters 9: 741–758. doi: 10.1111/j.1461-0248.2006.00924.x 16706917 10.1111/j.1461-0248.2006.00924.x

[43] Gower JC (1971) A general coefficient of similarity and some of its properties. Biometrics 27: 857–871.

[44] Laliberté E, Legendre P (2010) A distance-based framework for measuring functional diversity from multiple traits. Ecology 91: 299–305. 20380219 10.1890/08-2244.1

[45] Laliberté E, Shipley B (2010) FD: measuring functional diversity from multiple traits, and other tools for functional ecology. R package version 1.0–9. 10.1890/08-2244.1 20380219

[46] Bininda-Emonds ORP, Cardillo M, Jones KE, MacPhee RDE, Beck RMD, Grenyer R, et al (2007) The delayed rise of present-day mammals. Nature 446: 507–512. doi: 10.1038/nature05634 17392779 10.1038/nature05634

[47] Wilson DE, Reeder DAM (2005) Mammal species of the world: a taxonomic and geographic reference: Johns Hopkins University Press 2142 p.

[48] Kembel SW, Cowan PD, Helmus MR, Cornwell WK, Morlon H, Ackerly DD, et al (2010) Picante: R tools for integrating phylogenies and ecology. Bioinformatics 26: 1463–1464. doi: 10.1093/bioinformatics/btq166 20395285 10.1093/bioinformatics/btq166

[49] Schweiger O, Klotz S, Durka W, Kühn I (2008) A comparative test of phylogenetic diversity indices. Oecologia 157: 485–495. doi: 10.1007/s00442-008-1082-2 18566837 10.1007/s00442-008-1082-2

[50] Hijmans RJ, Cameron SE, Parra JL, Jones PG, Jarvis A (2005) Very high resolution interpolated climate surfaces for global land areas. International Journal of Climatology 25: 1965–1978.

[51] Andrews P, O'Brien EM (2000) Climate, vegetation, and predictable gradients in mammal species richness in southern Africa. Journal of Zoology 251: 205–231.

[52] Owen JG (1990) Patterns of mammalian species richness in relation to temperature, productivity, and variance in elevation. Journal of Mammalogy 71: 1–13.

[53] Ruggiero A, Kitzberger T (2004) Environmental correlates of mammal species richness in South America: effects of spatial structure, taxonomy and geographic range. Ecography 27: 401–417.

[54] Ruggiero A, Hawkins BA (2008) Why do mountains support so many species of birds? Ecography 31: 306–315.

[55] Mittelbach GG, Steiner CF, Scheiner SM, Gross KL, Reynolds HL, Waide RB, et al (2001) What is the observed relationship between species richness and productivity? Ecology 82: 2381–2396.

[56] Evans KL, Warren PH, Gaston KJ (2005) Species-energy relationships at the macroecological scale: a review of the mechanisms. Biological Review 80: 1–25. 10.1017/s1464793104006517 15727036

[57] Cusens J, Wright SD, McBride PD, Gillman LN (2012) What is the form of the productivity–animal-species-richness relationship? A critical review and meta-analysis. Ecology 93: 2241–2252. 23185885 10.1890/11-1861.1

[58] Willmott CJ, Matsuura K (2001) Terrestrial Water Budget Data Archive: Monthly Time Series (1950–1999). http://climate.geog.udel.edu/~climate/html_pages/README.wb_ts2.html.

[59] Foody GM (2005) Clarifications on local and global data analysis. Global Ecology and Biogeography: 99–100.

[60] Davies TJ, Buckley LB, Grenyer R, Gittleman JL (2011) The influence of past and present climate on the biogeography of modern mammal diversity. Philosophical Transactions of the Royal Society of London B: Biological Sciences 366: 2526–2535. doi: 10.1098/rstb.2011.0018 21807733 10.1098/rstb.2011.0018 PMC3138609

[61] Jetz W, Rahbek C, Lichstein JW (2005) Local and global approaches to spatial data analysis in ecology. Global Ecology and Biogeography: 97–98.

[62] Gouveia SF, Hortal J, Cassemiro FAS, Rangel TF, Diniz-Filho JAF (2013) Nonstationary effects of productivity, seasonality, and historical climate changes on global amphibian diversity. Ecography 36: 104–113.

[63] Keith SA, Kerswell AP, Connolly SR (2014) Global diversity of marine macroalgae: environmental conditions explain less variation in the tropics. Global Ecology and Biogeography 23: 517–529.

[64] Rangel TF, Diniz-Filho JAF, Bini LM (2010) SAM: a comprehensive application for Spatial Analysis in Macroecology. Ecography 33: 46–50.

[65] Janzen DH (1967) Why mountain passes are higher in the tropics. The American Naturalist 101: 233–249.

[66] Hedges LV, Olkin I (1985) Statistical methods for meta-analysis. New York: Academic Press.

[67] Rosenberg MS, Adams DC, Gurevitch J (2000) MetaWin: statistical software for meta-analysis. Sunderland: Sinauer Associates.

[68] Borcard D, Legendre P, Drapeau P (1992) Partialling out the spatial component of ecological variation. Ecology 73: 1045–1055.

[69] Loewenberg-Neto P (2014) Neotropical region: a shapefile of Morrone’s (2014) biogeographical regionalisation. Zootaxa 3802: 300–300. 10.11646/zootaxa.3802.2.12 24871011

[70] Morrone JJ (2014) Biogeographical regionalisation of the Neotropical region. Zootaxa 3782: 1–110. doi: 10.11646/zootaxa.3782.1.1 24871951 10.11646/zootaxa.3782.1.1

[71] Da Silva Cassemiro FA, De Souza Barreto B, Rangel TFL, Diniz-Filho JAF (2007) Non-stationarity, diversity gradients and the metabolic theory of ecology. Global Ecology and Biogeography 16: 820–822.

[72] Whittaker RJ, Nogués-Bravo D, Araújo MB (2007) Geographical gradients of species richness: a test of the water-energy conjecture of Hawkins *et al*. (2003) using European data for five taxa. Global Ecology and Biogeography: 76–89.

[73] Maestri R, Patterson BD (2016) Patterns of species richness and turnover for the South American rodent fauna. PloS one 11: e0151895 doi: 10.1371/journal.pone.0151895 26999278 10.1371/journal.pone.0151895 PMC4801412

[74] Tognelli MF, Kelt DA (2004) Analysis of determinants of mammalian species richness in South America using spatial autoregressive models. Ecography 27: 427–436

[75] Wheeler D, Tiefelsdorf M (2005) Multicollinearity and correlation among local regression coefficients in geographically weighted regression. Journal of Geographical Systems 7: 161–187.

[76] Fotheringham AS, Oshan TM (2016) Geographically weighted regression and multicollinearity: dispelling the myth. Journal of Geographical Systems 18: 303–329.

[77] Marquaridt DW (1970) Generalized Inverses, Ridge Regression, Biased Linear Estimation, and Nonlinear Estimation. Technometrics 12: 591–612.

[78] Tucker CM, Cadotte MW (2013) Unifying measures of biodiversity: understanding when richness and phylogenetic diversity should be congruent. Diversity and Distributions: 845–854.

[79] Davies TJ, Buckley LB (2012) Exploring the phylogenetic history of mammal species richness. Global Ecology and Biogeography 21: 1096–1105.

[80] Hoorn C, Wesselingh F, Ter Steege H, Bermudez M, Mora A, Sevink J, et al (2010) Amazonia through time: Andean uplift, climate change, landscape evolution, and biodiversity. science 330: 927–931. doi: 10.1126/science.1194585 21071659 10.1126/science.1194585

[81] Hawkins BA, McCain CM, Davies TJ, Buckley LB, Anacker BL, Cornell HV, et al (2012) Different evolutionary histories underlie congruent species richness gradients of birds and mammals. Journal of biogeography 39: 825–841.

[82] Safi K, Cianciaruso MV, Loyola RD, Brito D, Armour-Marshall K, Diniz-Filho JAF (2011) Understanding global patterns of mammalian functional and phylogenetic diversity. Philosophical Transactions of the Royal Society B: Biological Sciences 366: 2536–2544. 10.1098/rstb.2011.0024 PMC3138614 21807734

[83] Webb CO, Ackerly DD, McPeek MA, Donoghue MJ (2002) Phylogenies and community ecology. Annual Review of Ecology and Systematics 33: 475–505.

[84] Losos JB (2008) Phylogenetic niche conservatism, phylogenetic signal and the relationship between phylogenetic relatedness and ecological similarity among species. Ecology letters 11: 995–1003. doi: 10.1111/j.1461-0248.2008.01229.x 18673385 10.1111/j.1461-0248.2008.01229.x

[85] Wiens JJ, Graham CH (2005) Niche conservatism: integrating evolution, ecology, and conservation biology. Annual review of ecology, evolution, and systematics 36: 519–539.

[86] Pereira MJR, Palmeirim JM (2013) Latitudinal diversity gradients in New World bats: are they a consequence of niche conservatism? PLoS One 8: e69245 doi: 10.1371/journal.pone.0069245 23935963 10.1371/journal.pone.0069245 PMC3720615

[87] Pascual R (1984) Late Tertiary mammals of southern South America as indicators of climatic deterioration In: R J, editor. Quaternary of South America and Antarctic Peninsula. Rotterdam: Balkema Publisher pp. 1–30.

[88] Zurano JP, Martinez PA, Canto-Hernandez J, Montoya-Burgos JI, Costa GC (2017) Morphological and ecological divergence in South American canids. Journal of Biogeography 44: 821–833.

[89] Moura MR, Villalobos F, Costa GC, Garcia PC (2016) Disentangling the Role of Climate, Topography and Vegetation in Species Richness Gradients. PloS one 11: e0152468 doi: 10.1371/journal.pone.0152468 27014872 10.1371/journal.pone.0152468 PMC4807822

[90] Field R, Hawkins BA, Cornell HV, Currie DJ, Diniz Filho JAF, Guégan JF, et al (2009) Spatial species richness gradients across scales: a meta analysis. Journal of Biogeography 36: 132–147.

[91] Jenkins CN, Alves MAS, Uezu A, Vale MM (2015) Patterns of vertebrate diversity and protection in Brazil. PloS one 10: e0145064 doi: 10.1371/journal.pone.0145064 26679348 10.1371/journal.pone.0145064 PMC4682992

[92] Pascual R, Jaureguizar EO (1990) Evolving climates and mammal faunas in Cenozoic South America. Journal of Human Evolution 19: 23–60.

[93] Vizcaíno SF (2009) The teeth of the "toothless": novelties and key innovations in the evolution of xenarthrans (Mammalia, Xenarthra). Paleobiology 35: 343–366.

[94] Galewski T, Mauffrey J-F, Leite YL, Patton JL, Douzery EJ (2005) Ecomorphological diversification among South American spiny rats (Rodentia; Echimyidae): a phylogenetic and chronological approach. Molecular phylogenetics and evolution 34: 601–615. doi: 10.1016/j.ympev.2004.11.015 15683932 10.1016/j.ympev.2004.11.015

[95] Peixoto F, Villalobos F, Melo A, Diniz-Filho J, Loyola R, Rangel T, et al (2017) Geographical patterns of phylogenetic beta-diversity components in terrestrial mammals. Global Ecology and Biogeography 26: 573–583.

