# Supplementary material for: The latitudinal diversity gradient in South American mammals revisited using a regional analysis approach: The importance of climate at extra-tropical latitudes and history towards the tropics
Source: PLoS One. 2017 Sep 5;12(9):e0184057. doi: 10.1371/journal.pone.0184057 (PMC5584750; doi:10.1371/journal.pone.0184057)
Supplement: S1 File — (DOC) [file pone.0184057.s001.doc]

**S1 File. Null models to evaluate the association between phylogenetic diversity (AvPD), and taxonomic richness (TR) and observed spatial patterns of variation in AvPD and TR**

**Null models**

AvPD is calculated by averaging data on pairwise comparisons. We ran null models, separately for each mammal group, to verify that in our datasets the average variation in AvPD is independent of species richness variation. For each 110 km x 110 km cell, we fixed the observed TR values and estimated the mean and standard deviation of the AvPD values obtained after the shuffling of species names 10000 times on the phylogeny. The null models were done using the function ses.mpd () and the argument “taxa.labels” in R project package picante . We verified that mean random values of AvPD are not correlated with TR in our datasets (Fig. S1). The “funnel shape” in figure S1 is typical of phylogenetic pairwise measures (e.g., )

**Fig S1.** **The relationship between TR and observed and random AvPD for each mammal group.** Black empty circles: observed AvPD for each cell; red circles with error bars: mean random AvPD and standard deviation. AvPD: phylogenetic diversity, TR: taxonomic richness, r_null_: Coefficient of determination measuring the association between mean random AvPD and TR. The models were performed only for taxa analysed at species level.

**Spatial variation in phylogenetic diversity (AvPD) and taxonomic richness (TR)**

**Fig S2.** **Spatial variation in phylogenetic diversity (AvPD).** AvPD was the mean pairwise phylogenetic distance between all taxa in each cell; phylogenetic distance was the sum of the lengths of the branches connecting each pair of species (or genera in primates) based on the Bininda-Emonds et al. phylogeny. Rivers are drawn as brown lines and national borders are shown in grey. Maps are in Mollweide equal-area projection. The figure is reproduced from


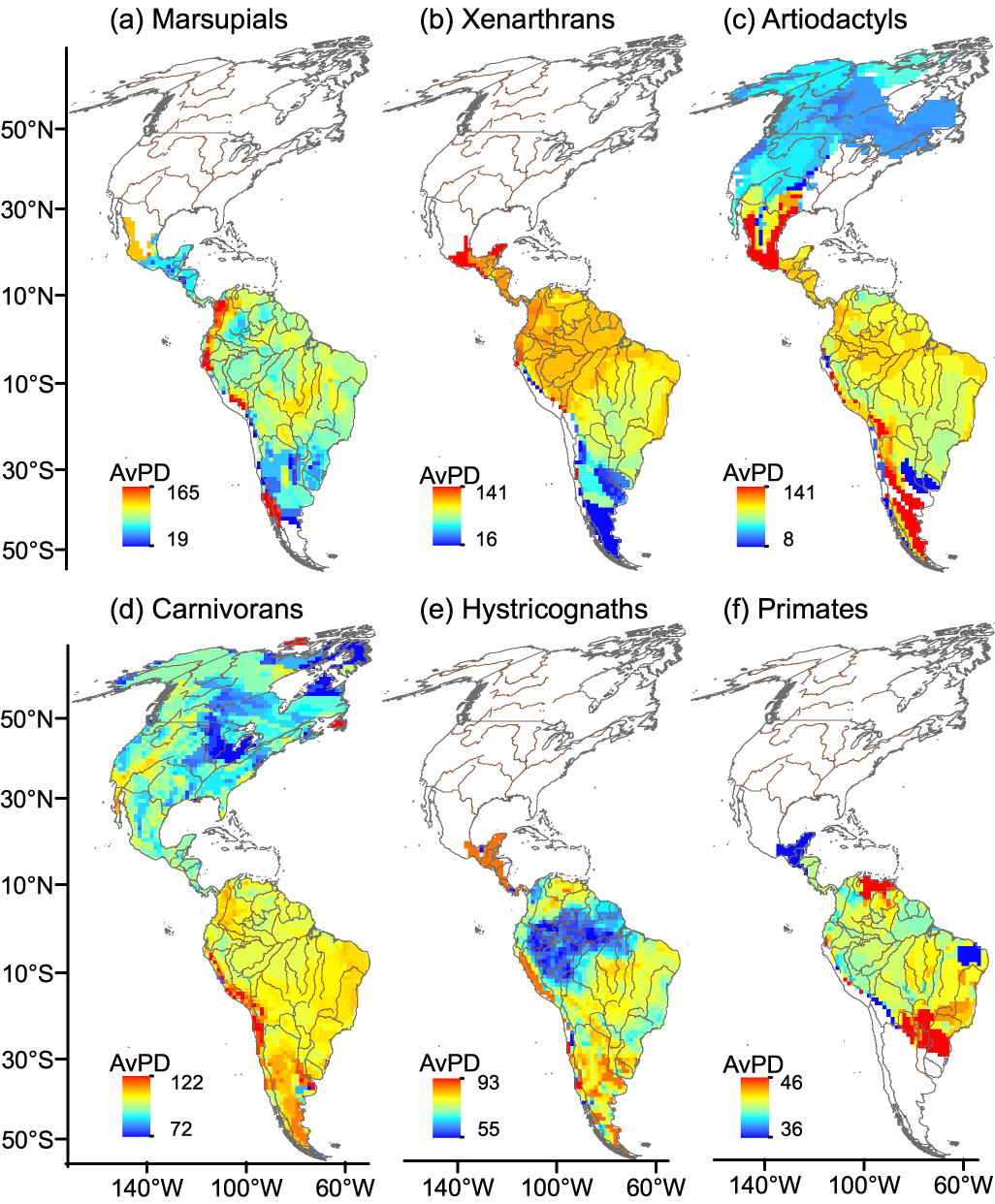


**Fig S3. Spatial variation in taxonomic richness (TR).** TR was calculated as the total number of species counted in each 110 x 110 km-cell of the grid map. Genera richness was calculated for primates. Rivers are drawn as brown lines and national borders are shown in grey. Maps are in Mollweide equal-area projection. The figure is reproduced from


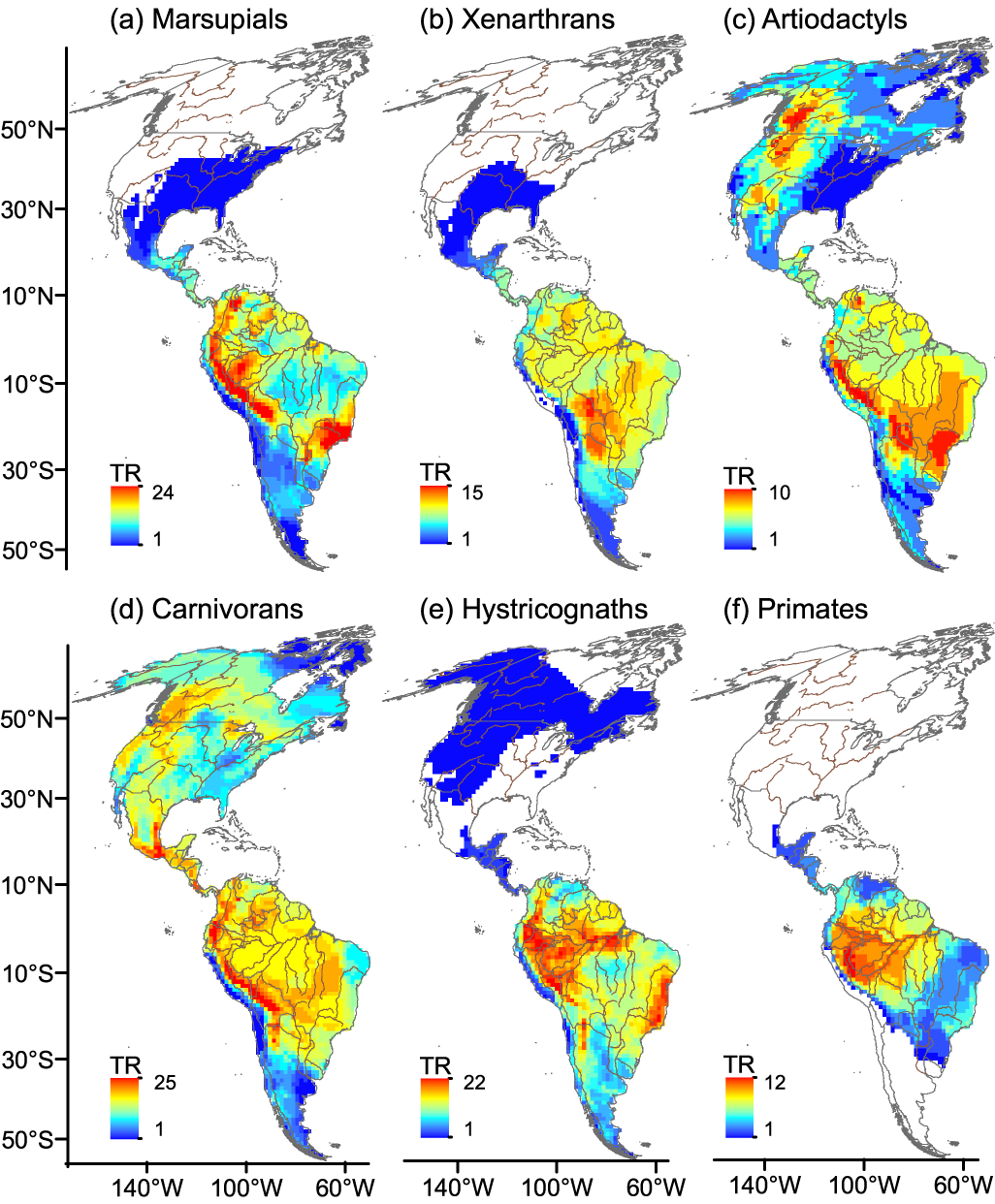


**References**

1. Kembel SW, Cowan PD, Helmus MR, Cornwell WK, Morlon H, et al. (2010) Picante: R tools for integrating phylogenies and ecology. Bioinformatics 26: 1463-1464.

2. Fritz SA, Rahbek C (2012) Global patterns of amphibian phylogenetic diversity. Journal of biogeography 39: 1373-1382.

3. Bininda-Emonds ORP, Cardillo M, Jones KE, MacPhee RDE, Beck RMD, et al. (2007) The delayed rise of present-day mammals. Nature 446: 507-512.

4. Fergnani PN, Ruggiero A (2015) Ecological diversity in South American mammals: their geographical distribution shows variable associations with phylogenetic diversity and does not follow the latitudinal richness gradient. PLoS ONE 10: e0128264. doi:0128210.0121371/journal.pone.0128264.
