# Supplementary material for: The latitudinal diversity gradient in South American mammals revisited using a regional analysis approach: The importance of climate at extra-tropical latitudes and history towards the tropics
Source: PLoS One. 2017 Sep 5;12(9):e0184057. doi: 10.1371/journal.pone.0184057 (PMC5584750; doi:10.1371/journal.pone.0184057)
Supplement: S2 File — (DOC) [file pone.0184057.s002.doc]

**S2 File. Slope (*b)* values and overall effect sizes of environmental variables and phylogenetic diversity (AvPD) on richness obtained for high R^2^ areas.**

**Table S1. Associations of taxonomic richness (TR) with environment and phylogenetic diversity (AvPD) obtained by GWR in areas where the models accounted for more than 50% of variation in taxonomic richness (= high R^2^ areas).** The numbers represent the mean and standard deviations (in brackets) of the local beta coefficients averaged over the total number of cells in each area. The geographic location of areas is shown in Fig 1 (main text). T = tropical, E = extra-tropical. Overall effect sizes (Fisher´s Z-transfrom, Zr: Hedges and Olkin ) were estimated using MetaWin v.2 . Beta coefficients greater than 1 were set at 0.99 for the calculations of effect sizes. R^2^ = coefficient of determination. TEMP = mean annual temperature, as surrogate for ambient energy; PREC = annual precipitation; ALTstd = standard deviation in elevation, NPP = net primary productivity, TEMPr = temperature range (maximum temperature of warmest month - minimum temperature of coldest month) and PRECcv = the coefficient of intra annual variation in precipitation, AvPD = phylogenetic diversity.

| *Tropical* | | | | | | | | |
| --- | --- | --- | --- | --- | --- | --- | --- | --- |
| R^2^ upper quartile areas | R^2^_adj_ | TEMP | PREC | ALTstd | NPP | TEMPr | PRECcv | AvPD |
| T1  n = 22 | 0.55  (0.00) | 0.11  (0.01) | -0.11  (0.00) | 0.08  (0.00) | 0.23  (0.00) | -0.29  (0.01) | -0.46  (0.01) | 0.13  0.01 |
| T2  (n = 21) | 0.55  (0.00) | 0.49  (0.07) | -0.18  (0.01) | 0.26  (0.01) | 0.49  (0.04) | 0.48  (0.15) | -0.39  (0.06) | -0.03  (0.01) |
| T3  (n = 125) | 0.57  (0.01) | -0.80  (0.07) | 0.07  (0.06) | 0.07  (0.09) | 0.36  (0.09) | -0.38  (0.10) | 0.21  (0.06) | -0.47  (0.11) |
| T4  (n = 11) | 0.64  (0.00) | 0.42  (0.00) | -0.09  (0.01) | -0.06  (0.01) | 0.23  (0.00) | -0.45  (0.03) | -0.11  (0.01) | -0.94  (0.04) |
| T5  (n = 26) | 0.65  (0.01) | 0.68  (0.01) | 0.14  (0.01) | 0.07  (0.02) | -0.02  (0.01) | 0.15  (0.02) | -0.11  (0.01) | -1.32  (0.05) |
| T6  (n = 264) | 0.53  (0.04) | 0.74  (0.14) | -0.16  (0.04) | 0.13  (0.02) | 0.15  (0.04) | 0.91  (0.25) | -0.06  (0.05) | -0.92  (0.11) |
| T7  (n = 12) | 0.70  (0.00) | -0.06  (0.01) | -0.20  (0.00) | 0.06  (0.00) | 0.20  (0.00) | 0.08  (0.02) | -0.36  (0.03) | -0.75  (0.02) |
| T8  (n = 73) | 0.71  (0.01) | 0.26  (0.03) | -0.06  (0.07) | 0.17  (0.02) | 0.31  (0.01) | 0.58  (0.04) | -0.32  (0.07) | -0.64  (0.02) |
| T9  (n = 221) | 0.72  (0.01) | -0.05  (0.17) | -0.31  (0.08) | 0.08  (0.12) | 0.20  (0.08) | -0.02  (0.11) | -0.05  (0.06) | -0.68  (0.03) |
| T10  (n = 46) | 0.72  (0.01) | -0.01  (0.01) | 0.04  (0.04) | -0.29  (0.01) | 0.14  (0.02) | 0.45  (0.07) | -0.52  (0.09) | -0.60  (0.05) |
| T11  (n = 112) | 0.72  (0.01) | 0.26  (0.06) | -0.05  (0.07) | -0.08  (0.05) | 0.28  (0.13) | 0.20  (0.24) | -0.32  (0.21) | -0.33  (0.11) |
| T12  (n = 75) | 0.73  (0.02) | 0.10  (0.06) | 0.47  (0.03) | -0.16  (0.09) | 0.06  (0.04) | -0.07  (0.01) | -0.09  (0.03) | 0.13  (0.01) |
| T13  (n = 65) | 0.73  (0.02) | 0.28  (0.02) | 0.31  (0.05) | 0.02  (0.02) | 0.13  (0.02) | -0.12  (0.07) | 0.05  (0.02) | -0.35  (0.02) |
| *Overall effect size*  (95% CI) | 1.14  (1.06;  1.19) | 0.20  (0.13;  0.27) | -0.06  (-0.12; 0.01) | 0.04  (-0.04; 0.12) | 0.21  (0.14; 0.28) | 0.18  (-0.28; 0.65) | -0.16  (-0.30;  -0.02) | -0.77  (-1.17;  -0.36) |
| *Q_total_*  (probability) | 8.06  (0.78) | 383.62  (<0.001) | 53.79  (<0.001) | 11.77  (0.46) | 11.18  (0.51) | 5.89  (0.92) | 12.32  (0.42) | 16.71  (0.16) |
| *Extra-tropical* | | | | | | | | |
| E1  (n = 207) | 0.64  (0.05) | 0.28  (0.04) | 0.39  (0.15) | 0.17  (0.05) | -0.05  (0.12) | -0.30  (0.07) | -0.09  (0.03) | 0.03  (0.03) |
| E2  (n = 327) | 0.80  (0.08) | 0.85  (0.13) | -0.25  (0.18) | 0.04  (0.04) | 0.07  (0.07) | -0.16  (0.16) | 0.11  (0.09) | 0.28  (0.07) |
| E3  (n = 197) | 0.50  (0.02) | 0.83  (0.14) | -1.15  (0.16) | 0.34  (0.09) | 0.43  (0.07) | -0.42  (0.19) | -0.39  (0.11) | 0.21  (0.23) |
| E4  (n = 53) | 0.48  (0.01) | 0.46  (0.07) | -0.89  (0.23) | 0.66  (0.16) | 0.00  (0.04) | -0.11  (0.13) | -0.01  (0.01) | -0.26  (0.02) |
| E5  (n = 64) | 0.51  (0.03) | 1.32  (0.05) | -0.35  (0.04) | 0.17  (0.01) | 0.49  (0.01) | -0.53  (0.20) | 0.14  (0.02) | 0.07  (0.05) |
| E6  (n = 280) | 0.63  (0.02) | 1.04  (0.07) | -0.24  (0.11) | 0.62  (0.18) | 0.44  (0.10) | 1.01  (0.32) | -0.26  (0.16) | 0.04  (0.03) |
| E7  (n = 507) | 0.67  (0.05) | 1.43  (0.12) | 0.12  (0.04) | 0.23  (0.03) | 0.31  (0.08) | 0.15  (0.24) | 0.16  (0.03) | -0.45  (0.07) |
| E8  (n = 67) | 0.75  (0.02) | 0.41  (0.02) | 0.06  (0.02) | 0.36  (0.03) | 0.10  (0.02) | 0.08  (0.05) | 0.06  (0.01) | -0.32  (0.06) |
| *Overall effect size*  (95% CI) | 1.10  (0.93; 1.27) | 1.45  (0.59; 2.31) | -0.54  (-1.36; 0.28) | 0.35  (0.13; 0.57) | 0.24  (0.06; 0.43) | 0.16  (-0.81; 1.13) | -0.04  (-0.24; 0.15) | -0.05  (-0.33; 0.24) |
| *Q_total_*  (probability) | 7.26  (0.40) | 7.27  (0.40) | 7.30  (0.40) | 7.11  (0.42) | 7.7  (0.36) | 5.56  (0.59) | 5.64  (0.58) | 4.55  (0.72) |

**References**

1. Hedges LV, Olkin I (1985) Statistical methods for meta-analysis. New York: Academic Press.

2. Rosenberg MS, Adams DC, Gurevitch J (2000) MetaWin: statistical software for meta-analysis. Sunderland: Sinauer Associates.
