# Supplementary material for: The latitudinal diversity gradient in South American mammals revisited using a regional analysis approach: The importance of climate at extra-tropical latitudes and history towards the tropics
Source: PLoS One. 2017 Sep 5;12(9):e0184057. doi: 10.1371/journal.pone.0184057 (PMC5584750; doi:10.1371/journal.pone.0184057)
Supplement: S3 File — (DOC) [file pone.0184057.s003.doc]

**S3 File. The mapping of tropical areas with high taxonomic richness and low ecological diversity (high TR-low ED) and high taxonomic richness and high ecological diversity (high TR-high ED), and variation in topographic and climatic heterogeneity.**

**Fig S4.** **Spatial variation in topographic heterogeneity represented by ALTstd (standard deviation of elevation) downloaded from WorldClim v. 1.4 overlapped with areas of high taxonomic richness and low ecological diversity (high TR-low ED), and high taxonomic richness and high ecological diversity (high TR-high ED) identified within tropical latitudes**. Grey circles: high TR-low ED; black circles: high TR-high ED. Maps are in Mollweide equal-area projection.

**Fig S5. Spatial variation in climatic variability represented by TEMPr (temperature range) downloaded from WorldClim v. 1.4 overlapped with areas of high taxonomic richness and low ecological diversity (high TR-low ED), and high taxonomic richness and high ecological diversity (high TR-high ED) identified within tropical latitudes**. Grey circles: high TR-low ED; black circles: high TR-high ED. Maps are in Mollweide equal-area projection.

**Fig S6.** **Spatial variation in climatic variability represented by PRECcv (coefficient of variation in precipitation) downloaded from WorldClim v. 1.4 overlapped with areas of high taxonomic richness and low ecological diversity (high TR-low ED), and high taxonomic richness and high ecological diversity (high TR-high ED) identified within tropical latitudes**. Grey circles: high TR- low ED; black circles: high TR- high ED. Maps are in Mollweide equal-area projection.

**References**

1. Hijmans RJ, Cameron SE, Parra JL, Jones PG, Jarvis A (2005) Very high resolution interpolated climate surfaces for global land areas. International Journal of Climatology 25: 1965-1978.
