# Supplementary material for: The latitudinal diversity gradient in South American mammals revisited using a regional analysis approach: The importance of climate at extra-tropical latitudes and history towards the tropics
Source: PLoS One. 2017 Sep 5;12(9):e0184057. doi: 10.1371/journal.pone.0184057 (PMC5584750; doi:10.1371/journal.pone.0184057)
Supplement: S4 File — (DOC) [file pone.0184057.s004.doc]

**S4 File. Evaluation of collinearity in geographically weighted regression (GWR) models.**

In Table S2 in File S4 we show that local variance inflation factors (VIF) obtained for each predictor variable were generally below the threshold of 10 . There were two exceptions; a low number of cells had VIF values for temperature range above 10 in artiodactyls and carnivorans (values ranged from 1.9 - 11.3 in artiodactyls and from 1.8 - 10.5 in carnivorans, Table S2 in S4 File). We mapped VIF values of temperature range in artiodactyls and carnivorans (Fig S7 in S4 File) to show that VIF values above 10 were few and were located outside high tropical R^2^ areas (compare Fig S7 in S4 File with Fig 1 in main text).

**Table S2.** **The range of local variance inflation factors (VIF) obtained for each 110 km x 110 km cell for each predictor variable of taxonomic richness.**

|  | TEMP | PREC | ALTstd | NPP | TEMPr | PRECcv | AvPD |
| --- | --- | --- | --- | --- | --- | --- | --- |
| Marsupials | 2.7 - 3.4 | 2.3 - 5.6 | 1.8 - 2.6 | 2.2 - 3.8 | 1.8 - 3.6 | 1.6 - 2.6 | 1.0 - 1.3 |
| Xenarthrans | 3.1 - 6.3 | 2.2 - 4.9 | 1.7 - 3.0 | 2.1 - 3.2 | 2.1 - 3.8 | 1.6 - 2.5 | 1.3 - 3.6 |
| Artiodactyls | 1.6 - 6.9 | 2.3 - 5.9 | 1.3 - 3.0 | 1.4 - 4.2 | 1.9 - 11.3 | 1.1 - 2.9 | 1.1 - 2.9 |
| Carnivorans | 1.3 - 2.8 | 1.4 - 4.2 | 1.6 - 5.5 | 2.5 - 5.5 | 1.8 - 10.5 | 1.1 – 2.9 | 1.0 - 1.7 |
| Hystricognaths | 2.5 - 3.6 | 2.4 - 5.7 | 1.8 - 2.9 | 2.3 - 4.1 | 2.3 - 3.8 | 1.6 - 3.0 | 1.2 - 2.4 |
| Primates | 2.7 - 3.2 | 2.2 - 3.2 | 1.7 - 2.7 | 2.0 - 3.1 | 1.7 - 2.6 | 1.6 - 2.4 | 1.0 - 1.6 |

TEMP = mean annual temperature, as surrogate for ambient energy; PREC = annual precipitation; ALTstd = standard deviation in elevation, NPP = net primary productivity, TEMPr = temperature range (maximum temperature of warmest month - minimum temperature of coldest month) and PRECcv = the coefficient of intra annual variation in precipitation, AvPD = phylogenetic diversity. VIF values were calculated using the GWmodel package in R project .

**Fig S7.** **Local variance inflation factors (VIF) obtained for temperature range (TEMPr) in artiodactyls and carnivores in geographically weighted regression (GWR) models.**


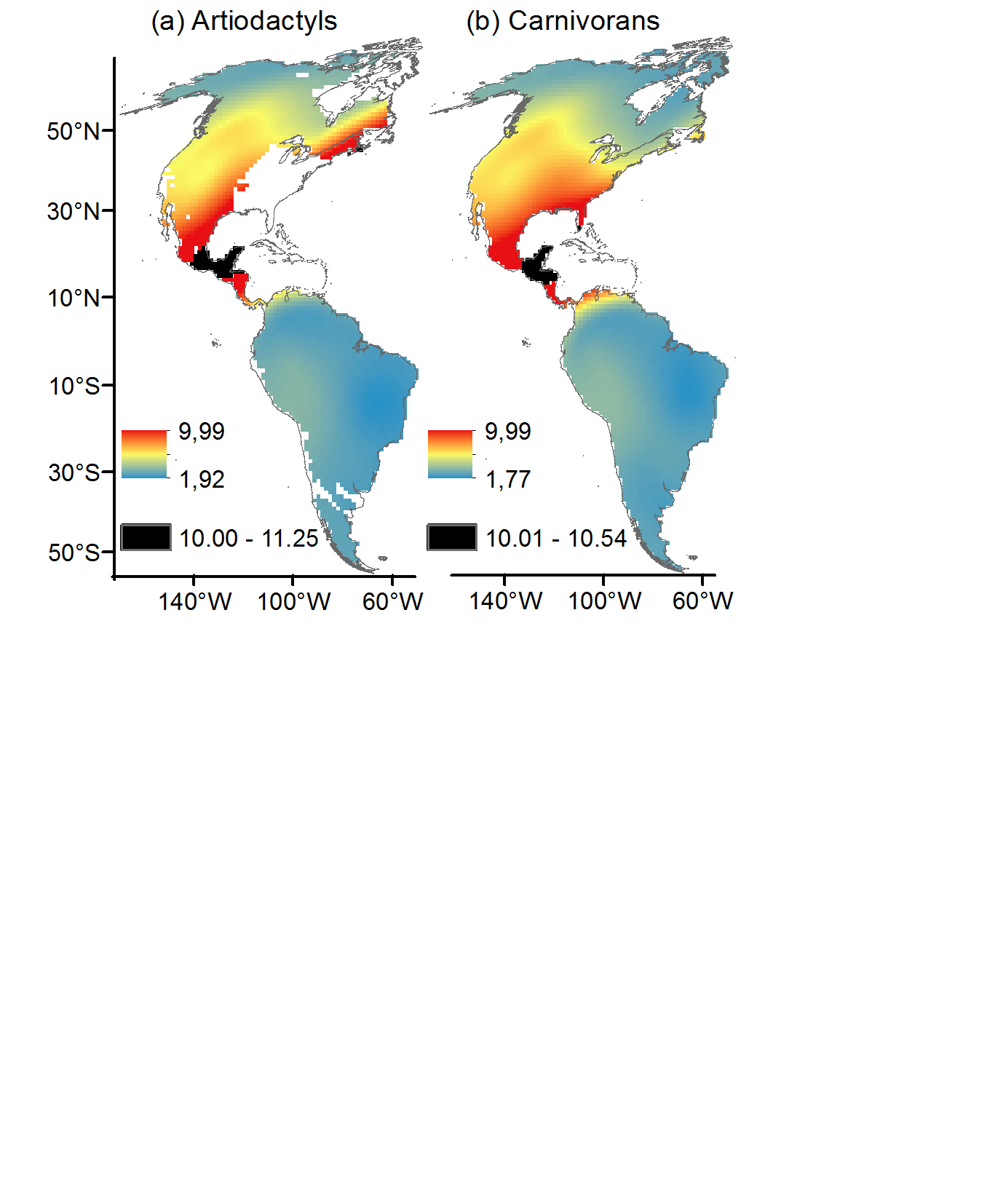


**References**

1. Marquaridt DW (1970) Generalized Inverses, Ridge Regression, Biased Linear Estimation, and Nonlinear Estimation. Technometrics 12: 591-612.

2. Gollini I, Lu B, Charlton M, Brunsdon C, Harris P (2013) GWmodel: an R package for exploring spatial heterogeneity using geographically weighted models. Journal of Statistical Software 63: 1-50.
